# Hemagglutination Detection with Paper–Plastic Hybrid Passive Microfluidic Chip

**DOI:** 10.3390/mi12121533

**Published:** 2021-12-09

**Authors:** Mahdee Samae, Surapong Chatpun, Somyot Chirasatitsin

**Affiliations:** Department of Biomedical Sciences and Biomedical Engineering, Faculty of Medicine, Prince of Songkla University, Hat Yai 90110, Thailand; dee.deeliver@hotmail.com (M.S.); surapong.c@psu.ac.th (S.C.)

**Keywords:** blood agglutination, xurographic technique, hybrid microfluidic chip, capillary-driven microfluidics, blood grouping

## Abstract

Hemagglutination is a critical reaction that occurs when antigens expressed on red blood cells (RBCs) react with the antibodies used for blood typing. Even though blood typing devices have been introduced to the market, they continue to face several limitations in terms of observation by the eye alone, blood manipulation difficulties, and the need for large-scale equipment, particularly process automated machines. Thus, this study aimed to design, fabricate, and test a novel hybrid passive microfluidic chip made of filter paper and polymer using a cost-effective xurography manufacturing technique. This chip is referred to as the microfluidic paper–plastic hybrid passive device (PPHD). A passive PPHD does not require external sources, such as a syringe pump. It is composed of a paper-based component that contains dried antibodies within its porous paper and a polymer component that serves as the detection zone. A single blood sample was injected into the chip’s inlet, and classification was determined using the mean intensity image. The results indicated that embedded antibodies were capable of causing RBC agglutination without a saline washing step and that the results could be classified as obviously agglutination or nonagglutination for blood typing using both the naked eye and a mean intensity image. As a proof-of-concept, this study demonstrated efficiency in quantitative hemagglutination measurement within a passive PPHD for blood typing, which could be used to simplify blood biomarker analysis.

## 1. Introduction

Blood agglutination or hemagglutination is a type of agglutination that occurs when antibodies bind to specific binding sites on antigens expressed on red blood cells (RBCs). Because of the ease of a clumping reaction, hemagglutination is a critical technique in blood type classification. Although blood typing discrepancies are uncommon, they must be a constant when patient safety is concerned [1,2]. To prevent this, using molecular blood grouping, such as PCR [3], next-generation sequencing [4], and whole-genome sequencing [5], could precisely provide more information on polymorphisms. However, these technologies are time-consuming, require central laboratory equipment, and are expensive. Therefore, hemagglutination for blood screening remains a major challenge for preventing blood typing discrepancies.

Hemagglutination tests have traditionally been conducted in two ways: manually using glass slides and tube agglutination tests, and automatically using a gel column test. The glass slide and tube tests are qualitatively interpreted by a skillful medical technician, making human errors a concern. Moreover, the tube test requires a blood sample of >1 mL [6], which cannot be obtained via minimally invasive techniques, such as finger pricking. In the automated gel column, the agglutination method requires a prepared blood sample with certain dilutions and requires highly trained personnel and sophisticated equipment. The solution to these issues is based on the concept of point-of-care testing (POCT), which features an easy to use, portable device, faster turnaround times, more rapid medical assessments in both qualitative and quantitative results, and the requirement of only a small volume of sample and reagent. Microfluidics are used in POCT to detect fluid manipulation in a single device with a small sample volume, enhancing the sensitivity and specificity for identifying target analytes, thereby overcoming those challenges [7].

There are many factors to be considered when developing POCT. Herein, two important factors are mentioned, i.e., material and fabrication techniques. Firstly, polymer and paper are two of the most frequently used microfluidic materials. Polymer materials provide various advantages, such as simple fabrication, cost-effectiveness, optical transparency, disposal, and suitability for prototyping [8]. However, protein absorption is the main drawback [9]. Meanwhile, paper chips are also low cost and have simple operation, good biocompatibility, distinct particle filtration, and passive flow control, yet are not optically transparent [10]. However, a paper-based hemagglutination platform requires a large amount of buffer to rinse the reaction to obtain a clear result [11,12]. The interpretation limitation of a paper-based chip can therefore be overcome by incorporating polymer materials that provide excellent optical clarity.

Secondly, microfabrication techniques for polymer [8], such as soft lithography, micro milling, injection molding, and laser cutting, are time consuming, complex, and require the use of expensive cleanroom facilities. Moreover, the techniques for paper-based microfluidics [13,14], such as hydrophobic-ink printing and masking, have a low resolution of patterns, a requirement for specific equipment and reagents. On the other hand, the xurographic technique [15,16,17] using both polymer and paper involves the use of a cutting plotter to create microchannels with the desired patterns, enabling the fabrication of inexpensive devices and materials in a short amount of time (a few minutes) without the need for robust devices and specific substances. Therefore, xurography could be chosen for microfabrication for both polymer and paper.

Researchers have reported microfluidic systems for blood agglutination. Many have proposed plastic microfluidic chips with a sophisticated flow control unit of fresh reagents [18,19,20]. Those required accurate micropumps and reagent solutions in control temperature containers before use, so that the operation could only be performed in a laboratory. Moreover, essential functions of a microfluidic chip are inextricably linked with the user’s experience on POCT. As using fresh reagent injection for hemagglutination detection on a chip is complicated, embedding dried reagents on the chip could reduce the number of steps required for use. Other research groups reported paper-based devices with fresh reagents and rinsing procedures before result interpretation [12,21,22]. Otherwise, false positive results could be detected. Therefore, the detection area of agglutination should be as clear as possible.

In this regard, we proposed a disposable microfluidic blood typing chip application, called the microfluidic paper–plastic hybrid passive device (PPHD), which provides a novel system for fluid handling and analysis for hemagglutination applications by using a blood grouping assay as a model. PPHD consists of an inlet, a reaction zone with reagent-embedded filter paper, a detection zone with transparent plastic, and an outlet. Blood samples would have capillary-driven flow into the device. Thus, in this study, we focused on the following aspects: (a) development of a quantitative paper–plastic-based microfluidic chip for blood agglutination application; (b) the amount of dried antibody molecules on the surface of cellulose fibers required to capture all RBCs via hemagglutination reaction; and (c) the effects of dried antibodies with sugars as stabilizers on the hemagglutination activity.

## 2. Materials and Methods

### 2.1. Fabrication of Paper–Plastic Hybrid Microfluidic Chip

**Figure 1:**
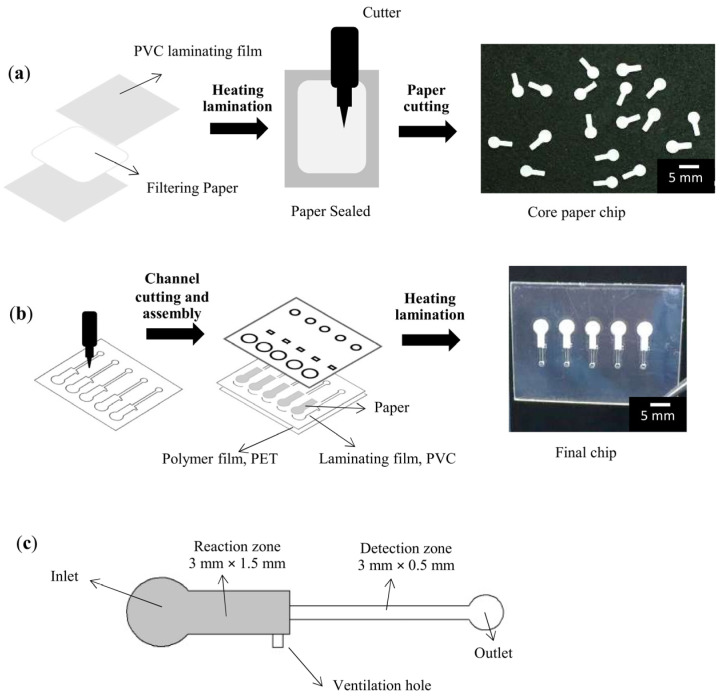
Fabrication processes of the PPHD: (**a**) preparation of the reaction zone on filtering core paper, (**b**) cutting the microchannel pattern and assembling the PPHD, and (**c**) schematic components of the PPHD. Scale bar of 5 mm.

The entire fabrication process was based on xurography with a cutting plotter (Graphtec cutting plotter CE6000-60, Graphtec, Shanghai, China). The xurographic process was used to directly cut the microfluidic patterns onto plastic substrates and filter paper (Whatman^®^ qualitative filter paper, Grade 4, Buckinghamshire, UK). Because the filter paper was directly damaged during the cutting process, it was thermally sealed with polyvinyl chloride (PVC) laminating films before cut, as shown in Figure 1a. All layouts were created using typical computer-aided design software and saved as the DXF files. Then, the files were imported into the Graphtec Studio software. Three layers of PPHD were presented: the upper, middle, and lower layers, as shown in Figure 1b. The upper layer was cut for the patterns of five inlets, ventilation holes, and outlets. The pattern of microchannels was carved on the middle layer, which was then adhered to the reaction zone with prepared filter paper (1.5 mm × 3 mm). The lower layer was obscured by polyethylene terephthalate film (PET: 25 mm × 35 mm × 0.125 mm). A roller thermal laminator (FGK-320 laminating machine, FGK, Zhejiang, China) was used to bond all three layers at 120 °C layer-by-layer, resulting in the final chip (25 mm × 35 mm × 0.5 mm). 

As shown in Figure 1c, PPHD comprises the following components: inlet (2 mm dia.), reaction zone (1.5 mm × 3 mm), ventilation hole (0.5 mm × 0.5 mm), detection zone (3 mm × 0.5 mm), and outlet (1.5 mm dia.). In details of the design of the microchannel, the inlet was wider than the reaction zone, as a sample reservoir. Next, the reaction zone comprised a ventilation hole and sudden contraction at the end of the zone, owing to control of the flow during the incubation period; meanwhile, the width of 1.5 mm was easy to handle and precisely cut by the plotter. The detection zone (0.5 mm) was wide enough to be visualized by the naked eye. The overall microchannels were 0.25 mm in depth, as the thickness of the filter paper used.

### 2.2. Reagents and Immobilized Reagent on Chip

The IgM antibody reagent solutions (anti-A, anti-B, anti-A,B, and anti-D) and blood samples (3% A-positive and B-positive standard red cell solutions) were obtained from the Thai Red Cross Society, Songkhla, Thailand, and the control solution of 0.1% sodium azide (NaN_3_) was used as a preservative reagent. The titers of the antibody reagents were 1:256, 1:256, 1:64, and 1:64, respectively. The concentrated reagent solutions were produced in a vacuum desiccator to obtain 2-fold (2×) and 3-fold (3×) concentrations of the initial solutions. Then, the immobilized reagents were embedded in the reaction zone of PPHD by drying 2 μL of the reagents overnight at room temperature in a vacuum desiccator. Three different sugars were used (purchased from Sigma-Aldrich, Singapore: sucrose, trehalose, and dextran in regular hydrogenated form).

### 2.3. Red Blood Cell Agglutination Assays

**Figure 2:**
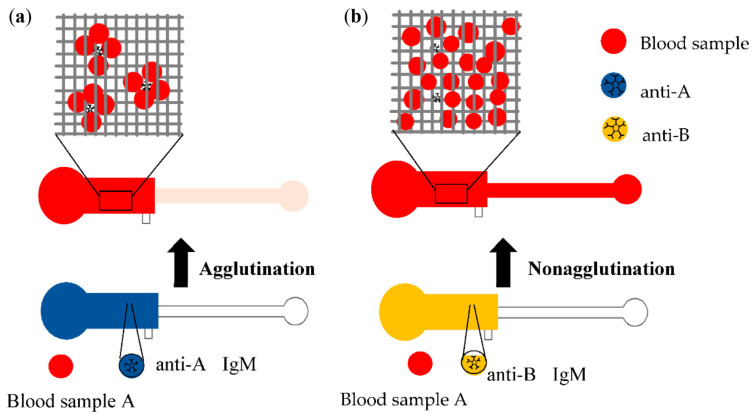
Schematic of the agglutination assay on PPHD. The clotting red cells of blood A cells and anti-A antibody (dark blue) cannot flow through mesh fibers of paper (**a**), while the anti-B antibody (yellow) does not react with A cells, leaving single red cells flow through the mesh (**b**).

Concentrated blood samples with a 45% hematocrit (HCT) were prepared using the 3% standard red cell solutions. The volume of 3 µL of the concentrated samples was deposited into each inlet of PPHD chips and incubated in the reaction zone at room temperature for 1 min. After opening the outlets, the sample flew into the detection zones via passive capillary force. As shown in Figure 2, a positive result of agglutination should reveal a well-defined colorless zone on the line detection zone, whereas nonagglutination, referred to as a negative assay, can easily be interpreted as the color of red blood on the line detection zone.

### 2.4. Quantification of Agglutination Assay

**Figure 3:**
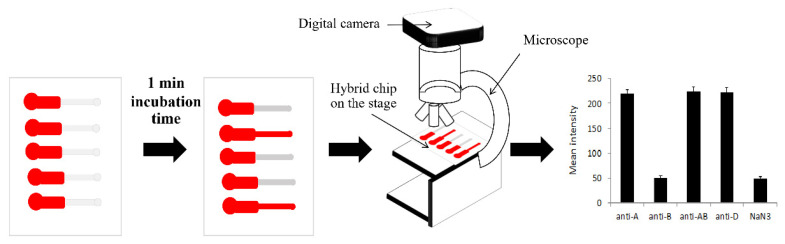
Schematic of the quantification of the agglutination assay process; the agglutination assay images were recorded using a digital camera via a microscope. Then, the agglutination assay images were analyzed, resulting in the grayscale mean intensity of the blood agglutination images.

Images of the agglutination assay on PPHD were captured using a Canon-5D-Mark-III digital camera attached to an optical inverted microscope with a 4× magnification objective lens (CKX41, Olympus, Tokyo, Japan) in bright field mode. The agglutination assay images were captured after passing the blood sample through the detection zone for 2 min. The images were first converted to 8-bit grayscale images, and the mean intensity of the region of interest (ROI) at detection zones was calculated, which was then analyzed using ImageJ software to invert and quantify the grayscale mean intensity of blood agglutination images (Figure 3). To avoid depicting a blood clotting feature, the ROI were 2 mm × 1.5 mm, 0.5 mm apart from the reaction zone, and 0.5 mm away from the outlet. All data in this study were presented as mean ± standard deviation (SD). *p* values < 0.05 were considered statistically significant. GraphPad Prism (GraphPad Software 8.0.2) was used for statistical analysis, ANOVA was used for comparisons in all analyses, and the quantification of the agglutination experiment was repeated six times.

### 2.5. Storage Stability Studies with Embedded Antibodies

Because antibodies are proteins produced by specific immune cells, they are critical. However, to avoid protein denaturation, the antibody must be freeze-dried, frozen in appropriate buffers, or refrigerated at 4 °C. The performance of the hemagglutination bioassay was affected by the storage and stabilization of embedded antibodies on PPHDs. In this study, all PPHDs were stabilized with three types of sugar: sucrose, trehalose, and dextran, which were blended with the antibody solutions at different concentrations of 5 and 10 (%*w*/*v*). The 3 µL of concentrated solutions were placed in the reaction zone and dried over in a vacuum desiccator at 25 °C. The PPHD was then vacuum sealed and stored away from light in a bag vacuum sealer. Quantitative agglutination detection was used to determine the residual bioactivity of the embedded antibody after five months of storage.

### 2.6. Hematocrit Variation and Incubation Time Study

The parameters of the antigen–antibody reaction were controlled for all experiments based on the factors affecting them, such as humidity and temperature. Consequently, only the effect of RBC concentration or hematocrit was studied. Various hematocrit levels were prepared in 1.5-mL Eppendorf tubes for separated red cells using a hematocrit centrifuge (Sigma 3-18KS, Osterode am Harz, Germany) at 1500 rpm during the RBC preparation. A microhematocrit reader was used to calculate the hematocrit of the blood samples. Hematocrit values for normal blood cell hematocrits range from 5 to 30, 40, 50, and 70%. The mean intensity of agglutination was calculated using 3 µL of various hematocrit blood samples. Furthermore, the incubation time for a completed agglutination reaction, particularly the agglutination between antigen and dried embedded-antibody based on PPHD, is an important parameter. The optimal incubation time for a completed agglutination reaction from 10–60 s was investigated in this study in relation to the dried antibody concentration, which was expressed in terms of “×” to indicate the initial concentration. In addition, 1× and 2× were 1 and 2 times the initial dried antibody concentration, respectively.

### 2.7. Statistical Analysis

The quantitative results of each experiment were repeated six times. GraphPad Prism 8.0.2 was used for statistical analysis. ANOVA was used for comparisons in all experiments. The data were presented as mean ± standard deviation (SD), and *p*-values < 0.05 were considered to be statistically significant. 

## 3. Results and Discussion

### 3.1. Image Observation of Blood Agglutination

**Figure 4:**
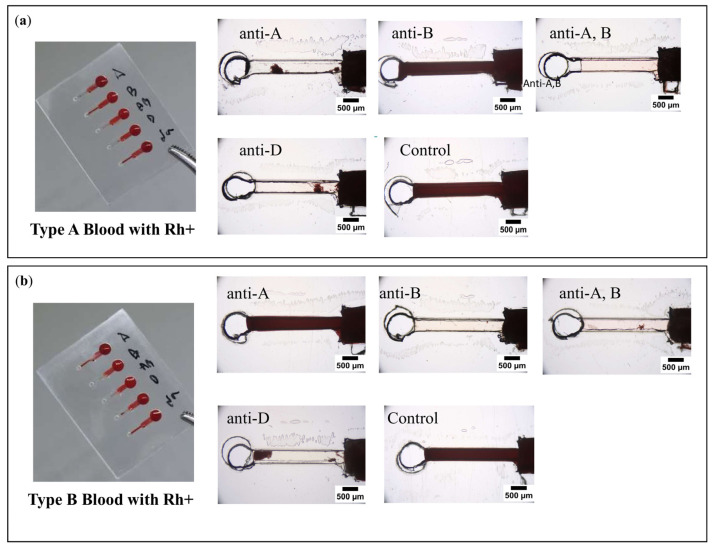
Representative features of blood agglutination and nonagglutination at the detection zone on PPHD test results are shown for (**a**) A with Rh+ and (**b**) B with Rh+ blood types. These visualized results are implied by the red lines and the corresponding A, B, A,B, D, and Na symbols on the top, which label the antibody locations.

Interestingly, the interpretation results were easily interpretable based on naked-eye observation. To determine blood agglutination and nonagglutination in PPHD, a simple interpretation of the blood typing experiment was performed to examine the images of each detection zone. The acquired images clearly differentiated between blood agglutination and nonagglutination, with blood agglutination being defined as clear blood at a detection zone with only a few spots of blood, whereas blood nonagglutination was defined as full blood at the detection zone (Figure 4). This distinction between A and B blood types and the recovery of dried antibodies of activity were confirmed following dissolution in blood samples. By examining the images of each detection zone, we were able to perform a simple direct interpretation of the blood typing assay in the PPHD experiment. Agglutination must occur during the hemagglutination reaction. When type A RBCs are exposed to anti-A, B, and anti-D, agglutination must occur. Similarly, type B RBCs react with anti-B, A, and anti-D. In contrast, when type A RBCs are exposed to anti-B and control solutions, most of the reactant solutions do not contain agglutinated blood cells. Moreover, significant blood collection may alter the rheological properties of the blood due to the formation of RBC or rouleaux aggregates that resemble agglutinates. The formation of rouleaux within the biochips has a negligible effect on some images at the detection zone, as shown in Figure 4. Not only the image observation as a quantitative result but also the naked-eye qualitative criteria requiring no prior experience could identify the blood agglutination on the chip. According to some studies, observing blood agglutination on a chip requires only quantitative results from skilled operators [23,24,25]. The naked-eye observation is an important interpretation because it is the first step to screening blood typing results.

### 3.2. Quantification of Blood Agglutination

**Figure 5:**
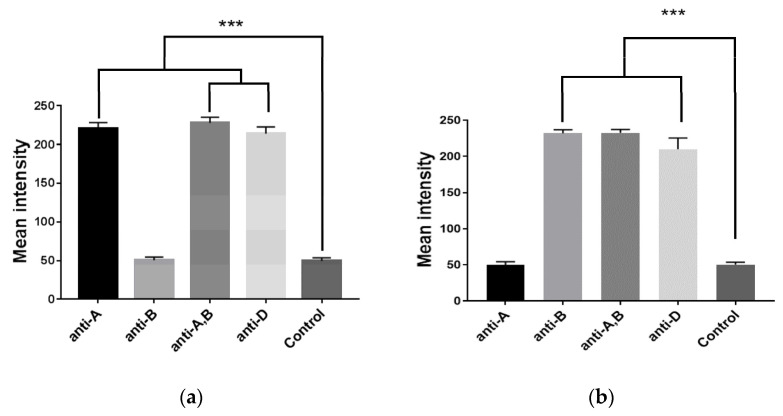
Mean intensity detection of (**a**) blood A Rh+ and (**b**) blood B Rh+; showing data sets that are significant at different levels: *** *p* ≤ 0.001.

The qualitative result of the hemagglutination assay was achieved via image processing of ROI digital pictures of the detection zone. Even though the width of the detection zone was 500 μm, which could be observed by the naked eye, it was unclear in some conditions, such as low light intensity, necessitating the use of digital image processing to eliminate the possibility of human errors in interpreting the assay result. To quantitatively determine the blood agglutination reaction, images of the detection zones were captured digitally and processed to obtain the image intensity. The pixel values ranged from 0 to 255 (darkest to brightest) in accordance with the 8-bit grayscale file format. Figure 5a,b demonstrate a highly statistically significant difference (*p*-value ≤ 0.001) in mean intensity between agglutination and nonagglutination reactions (220.5 ± 9.5 and 49.9 ± 4.0, respectively) that could also easily be classified to confirm both A and B blood types. According to the standard cell, which is normally characterized as a level 4+ strength of agglutination, this could be the cause of the complete red cell agglutination reaction plugging onto the pores of the filter paper, so that there is no outflow of incomplete reaction of red cells into the detection zone. However, previous work [26] has shown that the image processing technique was limited, as the strength of agglutination varied from level 0–4+. Another detection technique, such as impedimetric measurement, could be applied. Thus, it could clearly investigate blood typing with a strong agglutination reaction using a dried antibody on PPHD by comparing the mean intensity of the agglutination and nonagglutination reactions, due to the advantages of the paper-based reaction zone and the clear plastic-based detection one.

### 3.3. Immobilized Antibody Stability and Storage Time

**Figure 6:**
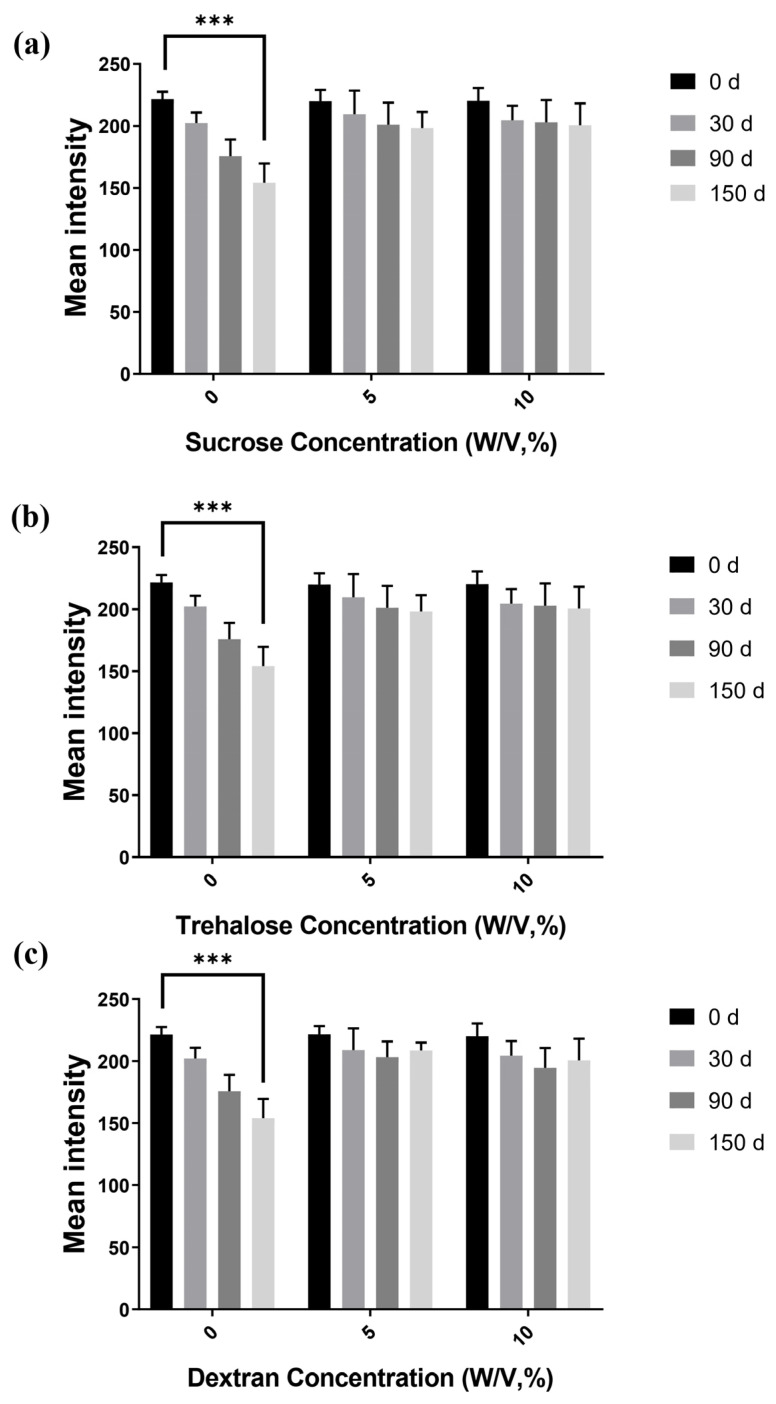
Immobilized-antibody storage stability with various sugar blending. Immobilized antibody with (**a**) sucrose, (**b**) trehalose, and (**c**) dextran. The mean intensity was calculated by comparing the blood agglutination of immobilized-antibody activity that remained after storage with the activity at day 0 for each group; *** *p* ≤ 0.001.

An important parameter in the development of the PPHD for blood typing with embedded antibodies is the potential antibody stabilizer, as protein instability on POCT devices severely limits commercial development. Although POCT devices with immobilized antibodies have been commonly used, the improved shelf life may allow for full storage on PPHD. The antibody and sugar were evaluated in this study under various conditions using the blood agglutination reaction (anti-A with blood sample A positive). Consequently, the mean intensity significantly decreased by at least 36% from 0 to 150 days of storage, especially in the absence of sugars. Nevertheless, when sugar was added, the mean intensity gradually decreased, resulting in a reduction of 9%, even on day 150 after the sugar was added, as shown in Figure 6. According to a study conducted by Cao et al. [27], the polysaccharide can sufficiently protect to engage in the interaction with dried antibodies within two months. Consequently, sugars were able to act as stabilizers for dried antibodies during long-term storage because disaccharides, such as trehalose and sucrose, could preserve both the structure and function of isolated proteins during drying. This ability of the disaccharide to stabilize proteins while drying resulted in the disaccharide forming hydrogen bonds with the proteins. Moreover, during dehydration and storage, antibodies combined with sugars can result in a reaction zone surface with a high viscosity and low molecular mobility, preventing proteins from denaturing [28]. Antibodies are prevented from unfolding when they are entrapped in dried sugars with dissimilar homogeneity. Thus, during storage, the entrapment of dried antibody in sucrose, trehalose, or dextran with good dispersion changed. In particular, dextran contains hydroxyl groups as its primary functional groups, making it extremely hydrophilic and uncharged, which may be advantageous for standard bioassays [29]. Further, the polysaccharide dextran [27] can interact with the antigen–antibody reaction, serving as an excellent model for studying the agglutination reaction [30]. Compared to a previous work where the dried-antibody reagents were immobilized on a plastic surface [17], this study reduced the amount of sugars, yet increased the value of the mean intensities, resulting in a low signal-to-noise ratio. Immobilizing the antibody with the stabilizers of sugars on the surface of cellulose fibers could improve the dissolving of antibody molecules and prolong the storage time at room temperature.

### 3.4. Effect of Hematocrit on Hemagglutination

**Figure 7:**
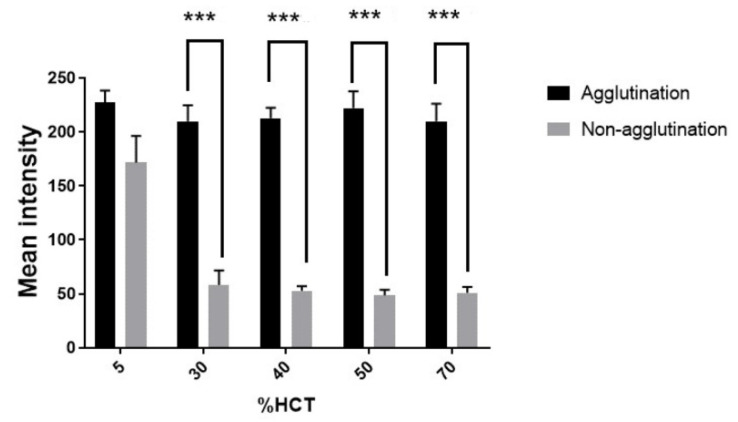
Comparison of blood agglutination and nonagglutination with various percentages of hematocrit (%HCT) using mean intensity images; highlighting data sets that are statistically significant at various levels; *** *p* ≤ 0.001.

A normal hematocrit ranges (%HCT) from 37 to 47% for women and 42 to 52% for men [31]. Other ranges can be used to diagnose certain conditions related to health status, such as dehydration (>70%) or anemia (<30%). Meanwhile, in a central laboratory, washed blood of 5% HCT is used. In this study, the effect of hematocrit variation on the agglutination assay of PPHD was therefore investigated under 5% and 30–70% HCT. In the range of 30–70%, there was a highly significant difference (*p*-value ≤ 0.001) in the mean intensity between the agglutination blood reaction at 213.4 ± 14.4 and nonagglutination at 52.5 ± 8.4. However, at 5% HCT, there was no significant difference between the agglutination and nonagglutination reactions (Figure 7). The sample with a low hematocrit level at 5% HCT could be affected by the prozone effect where the amount of antibody is too high compared to that of antigen. This implied that the concentration of antibody of PPHD could be suitably optimized for use in the blood bank laboratory. In contrast, blood with 30–70% HCT might be used for blood group screening bedside. However, other effects of substances in blood could be further studied, such as the amount of lipids, pathogens, fibrins, EDTA, and so on.

### 3.5. Effect of Incubation Time on Agglutination

Antigen–antibody manifest themselves as time-dependent effects. Reactants should be incubated for the optimal amount of time. If the incubation period is too short, the antigen and antibody reaction may not occur effectively. However, prolonged incubation may result in dissociation of antigen–antibody complexes [32]. Based on the traditional glass slide test of hemagglutination, whose incubation period between fresh antibody and whole blood is about 30–60 s, this study tracked the assay’s progress for completed agglutination reaction during 10–60 s related to 1× (initial concentration), 2× (2 times the initial dried antibody concentration), and 3× (3 times the initial dried antibody concentration).

**Figure 8:**
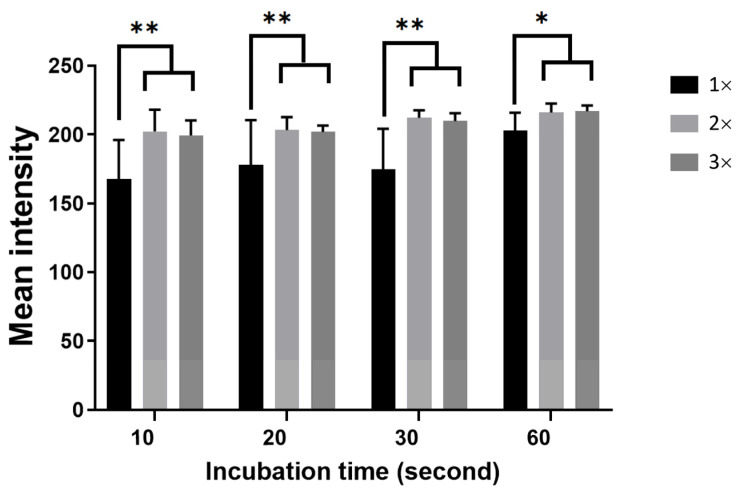
Comparison of the dried antibody concentration and different incubation times by mean intensity images; * *p* < 0.05, ** *p* < 0.01.

To control the incubation time, the ventilation hole and the reaction zone with sudden contraction were designed to stop the flow at the end of the zone. Consequently, the dried reagents dissolved and reacted with the blood sample solution. At a certain incubation time, the outlet was opened, resulting in the mixed solution flying in the detection zone via capillary effect. As shown in Figure 8, the longer the incubation time, the higher the mean intensity for the 1× concentration, reaching the maximum at 200. If the incubation time was shorter than 60 s, the red cells from the incomplete agglutination reaction could flow out to the detection zone, resulting in the intensity gradually decreasing lower than 200. However, for the concentrated 2× and 3×, the intensity remained almost constant over 200, significantly different from the initial concentration of 1×. This could imply that the sufficient agglutination reaction could reach 60 s for 1×, while only 20–30 s for 2×–3×. Moreover, the higher the dried-reagent concentration, the more reagents dissolved, resulting in better reaction completion. Compared to other reports [20,33,34], the proposed PPHD could therefore reduce time-to-result within 3 min without active pumps, fresh reagents, and a few steps of operation.

### 3.6. Limitations of this Study

This study investigated the agglutination reaction for proof-of-concept detection on a chip using standard red cell solutions for antibody screening tests in blood bank units. Therefore, there are some limitations to be considered in future works. Firstly, the blood sample was not whole blood from donors with EDTA; therefore, further study could investigate the effects of biomolecules in serum. Secondly, the strength of agglutination, according to the appearance of antigens on red cell membranes (level 0–4+), should be clarified. Finally, the validation with standard techniques, such as automated gel column testing and reproducibility performed by medical technologists, has to be evaluated before clinical trials.

## 4. Conclusions

The proposed fabrication process utilized a cost-effective xurography technique to combine PVC films and cellulose filter papers. The PPHD for the hemagglutination assay was quantitatively evaluated via the image processing parameter of mean intensity. The mean intensity of the agglutination reaction was four times higher than that of nonagglutination. Thus, the interpretation of the agglutination results was straightforward from a naked-eye perspective and also significantly different using image mean intensity. Moreover, storage conditions, antibody concentration, hematocrit, and incubation time were investigated. The agglutinated blood was filtered onto the paper substrate via specific antigen-antibody interaction, whereas the nonagglutinated blood was passively discharged into the detection zone via capillary action without rinsing buffers. Using blood typing as a model, the distinction between A and B blood types confirmed that dried antibodies of activity recovery were guaranteed following their dissolution in blood sample solutions. A more practical device would include preservation agents (sugars) dry on paper. When preservative sugars were added and stored at room temperature under ambient conditions for up to 150 days, no remarkable effect was observed (<9% intensity reduction). In the hematocrit range of 30–70%, PPHD was still applicable, yet at 5% HCT there was no significant difference between agglutination and nonagglutination results. The incubation time could provide the maximum performance after 30 s, resulting in a time-to-result for PPHD of only 3 min. Future works could consider whole blood from donors, and a standard procedure validation could be done.
